# Forecasting the Dialysis Burden in Japan: Validation‐Based Projections of Prevalence and Incidence Through 2050

**DOI:** 10.1002/1744-9987.70183

**Published:** 2026-07-06

**Authors:** Hatice Şahin, Berrak Itır Aylı, Nüket Paksoy

**Affiliations:** ^1^ Department of Nephrology Etlik City Hospital Ankara Türkiye; ^2^ University of Westminster London UK; ^3^ Department of Public Health Hacettepe University Faculty of Medicine Ankara Türkiye

**Keywords:** dialysis, disease projection, end stage renal disease, ensemble modeling, forecasting, Japan, time‐series analysis

## Abstract

**Background:**

Japan has one of the highest dialysis prevalence rates worldwide and a shrinking, aging population. Whether dialysis burden has entered a sustained post‐peak phase or whether recent declines partly reflect pandemic‐related disruptions remains uncertain.

**Methods:**

We analyzed nationwide data from the Japanese Society for Dialysis Therapy Renal Data Registry (1983–2022) to project dialysis prevalence and incidence through 2050. Seven forecasting approaches were implemented, including classical time‐series models, demographic regression models, machine‐learning algorithms, and a performance‐weighted ensemble. Models were trained on 1983–2017 data and validated against a holdout period (2018–2022) encompassing the historical peak in dialysis prevalence in order to forecast accuracy using RMSE, MAE, and MAPE.

**Results:**

Dialysis prevalence increased from 52 505 patients in 1983 to a peak of 349 700 in 2021, followed by a decline in 2022, though the contribution of COVID‐19‐related excess mortality to this inflection cannot be excluded. During validation, the ensemble model demonstrated the highest predictive accuracy for prevalence and incidence (MAPE 0.60% and 0.48% respectively). Individual models produced divergent long‐term trajectories, ranging from substantial decline to continued growth. The ensemble model projected a gradual decrease in dialysis prevalence to approximately 322 000 patients by 2050 (−7.2% vs. 2022) and a 24.4% reduction in annual dialysis incidence.

**Conclusions:**

Dialysis prevalence in Japan shows signs of a potential shift toward a downward trend with future burden increasingly driven by demographic change rather than rising incidence. Ensemble forecasting grounded in empirical validation provides balanced projections and highlights substantial uncertainty across modeling paradigms. Japan's experience offers important insights for other aging societies planning future kidney care services.

## 1 Introduction

End stage renal disease (ESRD) requiring renal replacement therapy (RRT) remains a major and growing public health challenge worldwide, driven by population aging and the rising prevalence of diabetes and hypertension [1, 2, 3]. Japan represents a particularly important case study for long‐term dialysis epidemiology. It has one of the highest dialysis prevalence rates globally, an exceptionally aged dialysis population, and a uniquely comprehensive national registry with high participation rates extending over several decades [2, 3, 4, 5].

The Japanese Society for Dialysis Therapy Renal Data Registry (JRDR) has provided uninterrupted nationwide surveillance since the early 1980s, enabling detailed evaluation of trends in dialysis incidence, prevalence, mortality, and patient characteristics [4, 5]. These data have underpinned several landmark analyses of dialysis epidemiology and forecasting. Most notably, Nakai et al. projected in 2012 that the Japanese dialysis population would peak around 2021 at approximately 350 000 patients, followed by a gradual decline driven by population contraction and aging [6]. The subsequent observation of a peak in 2021 and an initial decline in 2022 provides a rare opportunity to reassess long‐term dialysis projections using contemporary data and modern modeling approaches.

Globally, studies describing dialysis and kidney replacement therapy trends consistently highlight the central role of demographic change, particularly population aging and declining birth rates, in shaping future service demand in high‐income countries [1, 2, 3]. Registry‐based analyses from Europe, the United States, and Japan show that dialysis prevalence increasingly reflects improvements in patient survival and shifts in age structure, rather than continued growth in incidence alone [4, 7, 8]. Although several national and regional reports include short‐ to medium‐term projections of dialysis burden, these forecasts are typically based on single modeling approaches or descriptive trend extrapolation [9, 10]. As a result, long‐term dialysis projections remain an underexplored area despite rigorous records from a number of counties; projection sensitivity to underlying assumptions, direct comparisons between classical time‐series models, demographic regression frameworks, and machine‐learning approaches within national datasets remain uncommon in the literature.

Against this background, the present study aimed to project dialysis prevalence and incidence in Japan through 2050 using four decades of JRDR data and a comprehensive set of forecasting models, including classical time‐series approaches, demographic regression models, machine‐learning algorithms, and a performance‐weighted ensemble. By explicitly validating models against the recent structural turning point in dialysis prevalence (2018–2022), this analysis seeks to provide robust, policy‐relevant projections while quantifying uncertainty across competing modeling paradigms.

## 2 Methods

### 2.1 Study Design and Data Sources

This retrospective observational study utilized data from the Japanese Society for Dialysis Therapy Renal Data Registry (JRDR) spanning 1983 to 2022, representing 40 years of continuous dialysis surveillance data [4]. The JSDT facility survey achieves consistently high response rates (> 98% annually) and provides annual counts of prevalent patients, incident patients, and deaths at the facility level. We acknowledge that response rates, while high, are not 100% and may vary slightly across years; however, given the consistently high participation rate, this limitation is unlikely to substantially affect trend estimation.

We extracted annual data on prevalent dialysis patients (total patients receiving dialysis at year‐end), incident patients (new patients initiating dialysis during the year), patient deaths, and median age of both prevalent and incident populations. Population data for 1983–2022 and population projections (medium variant) for 2023–2050 were sourced from the United Nations, Department of Economic and Social Affairs, Population Division's records [11].

### 2.2 Statistical Analysis

#### 2.2.1 Model Development

We implemented seven forecasting models to project dialysis prevalence and incidence from 2023 to 2050:

- Autoregressive Integrated Moving Average (ARIMA): A univariate time series model that captures temporal autocorrelation patterns. Model parameters (*p*, *d*, *q*) were automatically selected using the Akaike Information Criterion (AIC).
- Exponential Smoothing State Space (ETS): A model that decomposes time series into error, trend, and seasonal components. The optimal model specification was determined automatically based on AIC.
- ARIMA with Exogenous Variables (ARIMAX): An extension of ARIMA incorporating total population and median population age as external regressors to account for demographic transitions.
- Dynamic Linear Regression Model (DLM): A time series linear model including trend, population, and median age as predictors, allowing the relationship between dialysis prevalence and demographic factors to vary over time.
- Polynomial Regression: A third‐degree polynomial model with population and median age covariates to capture potential non‐linear temporal patterns.
- Random Forest Regression: A machine learning ensemble method using 500 decision trees with year index, population, and median age as features.
- Extreme Gradient Boosting (XGBoost): A gradient boosting algorithm with regularization, configured with learning rate of 0.1, maximum depth of 4, and 100 boosting rounds.
- Ensemble Model: A weighted average of individual model forecasts, with weights proportional to the inverse of each model's Mean Absolute Percentage Error (MAPE) during validation. Several ensemble models were tested during modeling; best results were obtained when all models were included, and these final ensemble models were presented in the study. The exact weights of the final ensemble model have been presented in Table S1.

We included Random Forest and XGBoost to provide a comprehensive comparison across modeling paradigms, recognizing that the small annual sample size (*n* = 35 training observations) poses a substantial risk of overfitting for complex non‐linear algorithms. To mitigate this, Random Forest was configured with a large number of trees (500) to stabilize variance, while XGBoost incorporated L1/L2 regularization (default parameters), a conservative learning rate (0.1), and limited tree depth (max_depth = 4). No extensive hyperparameter search was performed because the training set was too small to support a reliable cross‐validation grid search without data leakage.

Notably, total population and median population age serve as proxy variables rather than direct causal drivers of dialysis burden. These covariates implicitly aggregate several distinct mechanisms, including population aging effects, changes in dialysis initiation thresholds over time, and shifts in the underlying composition of ESRD causes (e.g., diabetic versus non‐diabetic nephropathy).

#### 2.2.2 Model Validation

Models were trained on data from 1983 to 2017 (*n* = 35 years) and validated on the holdout period 2018–2022 (*n* = 5 years). This validation period was strategically selected to include the historical peak in dialysis prevalence (2021) and the first observed decline (2022), representing a critical structural change in the dialysis trajectory.

#### 2.2.3 Sensitivity Analysis for COVID‐19 Effects

Given that the COVID‐19 pandemic significantly affected dialysis patient dynamics in Japan from 2020 onward—with reported increases in mortality and alterations in incidence patterns—we performed a sensitivity analysis to assess whether model performance differed between the pre‐pandemic (2018–2019) and pandemic‐affected (2020–2022) portions of the validation period. This analysis evaluated whether the observed model rankings remained robust when validation was restricted to pre‐COVID data only. The full analysis results have been provided in Supporting Information.

#### 2.2.4 Performance Metrics

Model performance was evaluated using Root Mean Square Error (RMSE), Mean Absolute Error (MAE), Mean Absolute Percentage Error (MAPE), and *R* ^2^. We prioritize MAPE as our primary criterion for model selection, while reporting *R* ^2^ as a supplementary indicator. In time‐series forecasting applications with short validation windows and structural inflection points, *R* ^2^ can become misleading because it penalizes deviations from the validation‐period mean, which may not represent a stable benchmark. Negative *R* ^2^ values therefore indicate that predictions deviated from the observed mean more than a naïve constant prediction would but do not necessarily imply that the model lacks predictive utility. MAPE, by contrast, quantifies the average proportional error of each prediction relative to the corresponding observed value, making it more interpretable and robust in small‐sample validation settings.

After validation, all models were retrained using the complete 1983–2022 dataset to generate forecasts through 2050. All analyses were performed using R version 4.3.0 with the forecast, caret, randomForest, and xgboost packages.

## 3 Results

### 3.1 Descriptive Statistics

Between 1983 and 2022, the prevalent dialysis population in Japan increased from 52 505 to 347 474 patients, representing a 6.6‐fold increase over 40 years. The dialysis population grew continuously until reaching a peak of 349 700 in 2021, followed by a decline to 347 474 in 2022 (decrease of 2226 patients, −0.6%), although the extent to which this reflects a genuine structural turning point versus pandemic‐related excess mortality remains to be established. The median age of prevalent patients increased from 49.5 years in 1983 to 69.9 years in 2022.

### 3.2 Model Validation Performance

Table 1 and Figure 1 present the validation performance metrics for all models during the 2018–2022 holdout period.

**TABLE 1:** Prevalence model validation performance (2018–2022).

Model	RMSE	MAE	MAPE (%)	*R* ^2^
Ensemble	2142	2071	0.60	0.58
ETS	5537	3600	1.03	−1.63
DLM	11 055	8609	2.47	−9.47
ARIMAX	15 694	12 557	3.61	−20.11
Polynomial	13 884	13 069	3.77	−15.52
ARIMA	16 219	13 477	3.88	−21.44
Random Forest	36 338	33 868	9.76	−77.07
XGBoost	40 017	36 688	10.57	−286.29

*Note:* Negative *R* ^2^ values are expected in short validation windows containing structural turning points. In this setting, *R* ^2^ compares model predictions against the validation‐period mean, which is an unstable benchmark when the outcome reverses direction. MAPE provides a more informative measure of predictive accuracy and was used as the primary model selection criterion.

Abbreviations: MAE, mean absolute error; MAPE, mean absolute percentage error; *R* ^2^, coefficient of determination; RMSE, root mean square error.

**FIGURE 1:**
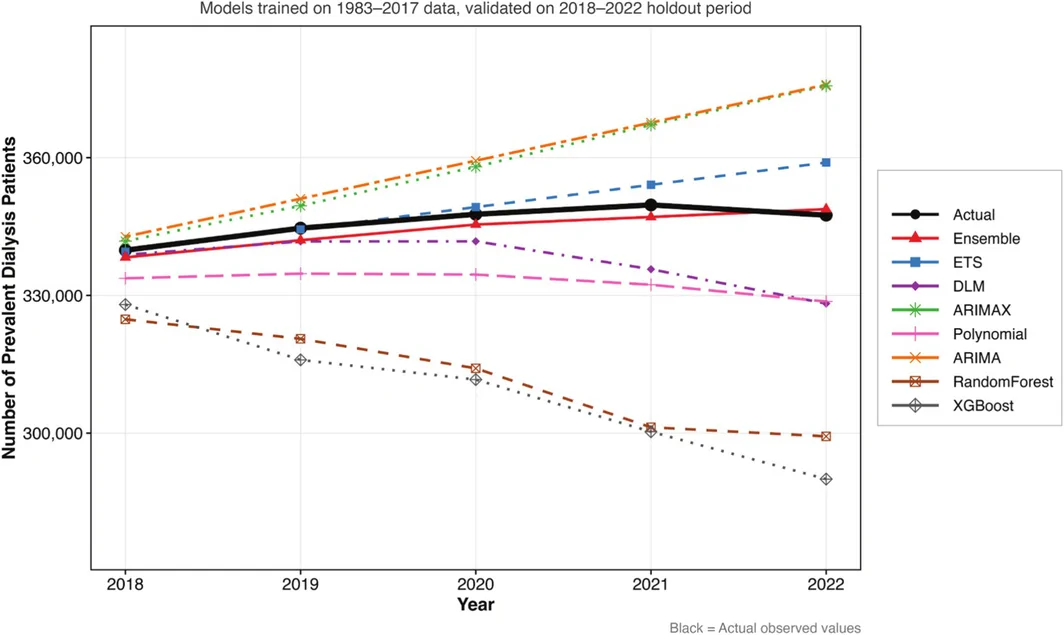
Prevalence model validation performance (2018–2022).

The ensemble model demonstrated superior predictive accuracy with a MAPE of 0.60%, substantially outperforming all individual models. Among individual models, ETS achieved the best performance (MAPE 1.03%), followed by DLM (2.47%) and ARIMAX (3.61%). Machine learning models (Random Forest and XGBoost) demonstrated suboptimal performance.

Model fit during the validation period, as measured by *R* ^2^, revealed substantial differences across approaches. The Ensemble model achieved a positive *R* ^2^ of 0.58, indicating that it explained approximately 58% of the variance in observed prevalence during the validation window. In contrast, all individual models produced negative *R* ^2^ values, ranging from −1.63 (ETS) to −286.29 (XGBoost). However, these negative values should be interpreted with caution: in time‐series forecasting over short validation windows (*n* = 5) that include structural inflection points, *R* ^2^ is inherently unstable and may be misleading. Negative *R* ^2^ indicates that model predictions deviated more from observed values than a simple mean‐based benchmark would, but does not necessarily imply fundamental model inadequacy. Given these limitations, we emphasize MAPE as the primary metric for model comparison, while presenting *R* ^2^ as Supporting Information.

### 3.3 Sensitivity Analysis: Impact of COVID‐19 on Model Performance

To assess the potential impact of COVID‐19 on validation results, we compared model performance between the pre‐pandemic period (2018–2019) and the pandemic‐affected period (2020–2022). During the pre‐COVID validation window, individual model performance was substantially better than during the COVID‐affected years, while the Ensemble model maintained stable performance across both periods.

For prevalence, ETS achieved a MAPE of 0.09% in 2018–2019 compared to 1.67% during 2020–2022, while the Ensemble model showed remarkable stability (0.71% pre‐COVID vs. 0.62% during COVID). The ARIMA model exhibited the largest performance degradation (+4.19 percentage points), reflecting its inability to capture pandemic‐induced structural changes without external covariates. Notably, the Ensemble model was the only approach that showed improved or stable performance during the pandemic period, likely because its inverse‐MAPE weighting naturally down‐weighted poorly performing models.

For incidence, similar patterns were observed: the Ensemble model maintained the lowest MAPE across both periods (0.34% pre‐COVID, 0.57% during COVID), while ARIMA showed the largest degradation (+6.38 percentage points). The relative ranking of models remained consistent across validation windows, with Ensemble and covariate‐incorporating models (ARIMAX) consistently outperforming pure time‐series approaches. Full sensitivity analysis results are presented in Tables S2 and S3 and illustrated in Figures S1 and S2.

These findings suggest that while COVID‐19 introduced structural discontinuities that degraded individual model performance, the Ensemble approach effectively mitigated these effects by adaptively weighting model contributions based on validation performance.

### 3.4 Dialysis Prevalence Projections to 2050

Table 2 presents projected dialysis prevalence at key milestone years using the ensemble and selected individual models, and Figure 2 illustrates the full trend.

**TABLE 2:** Projected prevalence (2025–2050).

Year	ARIMA	ETS	ARIMAX	DLM	Ensemble
2025	354 582	354 681	347 889	329 266	340 078
2030	362 301	362 223	351 114	323 810	342 443
2035	370 020	371 765	349 253	340 285	340 264
2040	377 740	380 308	339 938	375 305	336 805
2045	385 459	388 850	319 724	433 041	331 352
2050	393 178	397 393	295 506	499 625	322 454

**FIGURE 2:**
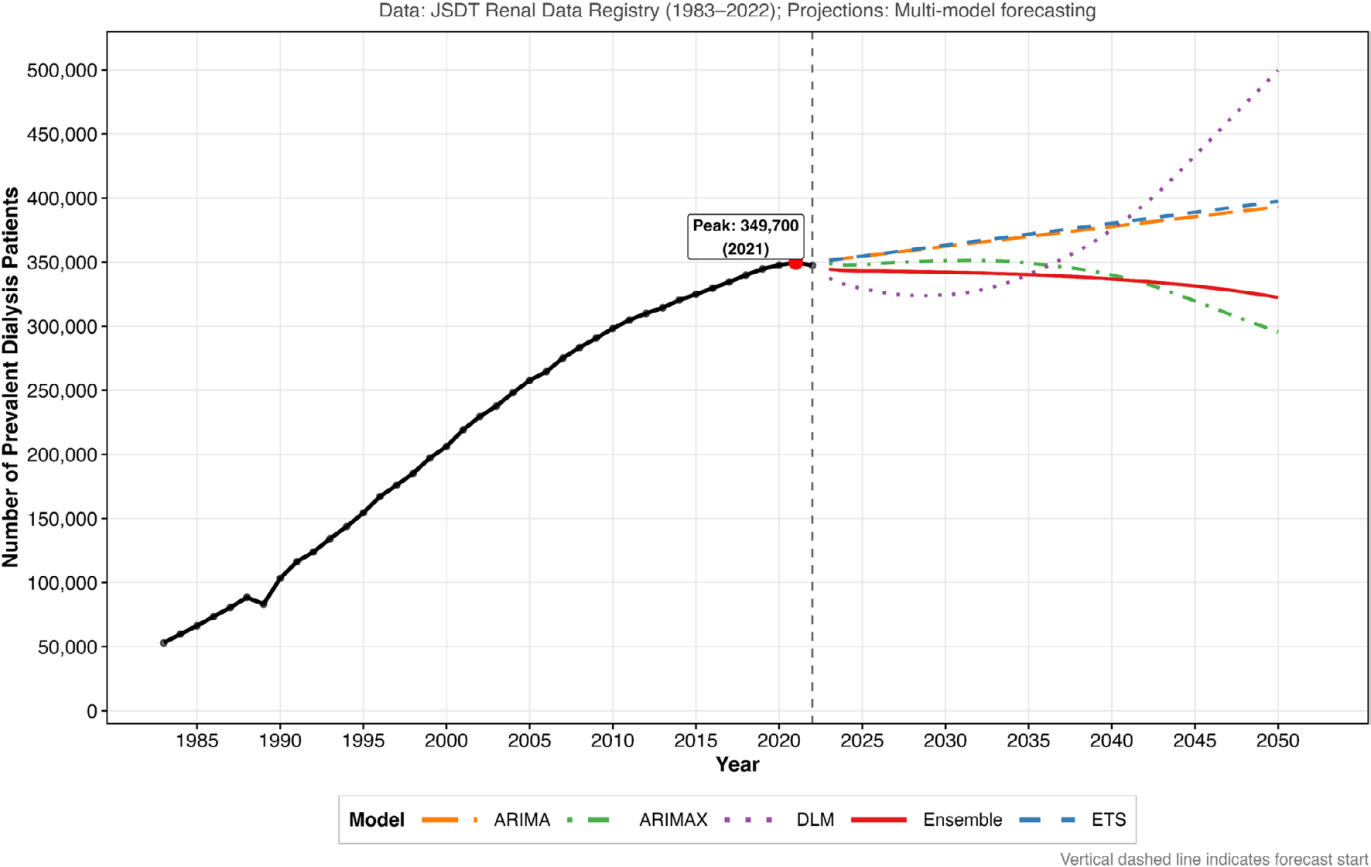
Projected prevalence to 2050.

The individual models yielded divergent long‐term trajectories. The ARIMAX model, incorporating population decline and aging as covariates, projected a peak around 2030 followed by sustained decline to 295 506 patients by 2050, representing a 15% decrease from 2022 levels. The ETS model projected continued linear growth, reaching 397 393 patients by 2050 (14% increase). The DLM projected accelerating growth driven by population aging effects, reaching 499 625 patients by 2050 (44% increase). The ensemble model, which achieved the best validation performance, projected a gradual decrease to 322 454 patients by 2050, a 7.2% decrease from 2022 levels. This projection is conditional on the assumption that post‐pandemic dynamics return broadly to the underlying pre‐COVID trajectory.

Inverse‐MAPE weighting for the prevalence ensemble assigned contributions of 41.1% to ETS, 17.1% to DLM, 11.7% to ARIMAX, 11.2% to Polynomial, 10.9% to ARIMA, with minimal contributions from Random Forest (2.6%) and XGBoost (5.4%). The Polynomial model has not been illustrated in Figure 2 and Table 2 due to the model projecting numbers below zero from 2044 onwards.

### 3.5 Model Agreement and Forecast Uncertainty

The divergence among model projections increased substantially over the forecast horizon. For 2030, the range of projections spanned from 323 810 (DLM) to 362 223 (ETS), a difference of approximately 38 000 patients. By 2050, this range widened considerably, from 295 506 (ARIMAX) to 499 625 (DLM), reflecting fundamentally different assumptions about how demographic changes will influence dialysis demand. The ensemble model, which weighted predictions according to validation performance, provided projections that fell within the middle range of individual model estimates.

### 3.6 Dialysis Incidence Trends and Projections

During the validation period (2018–2022), observed annual dialysis incidence in Japan declined modestly from 40 468 new patients in 2018 to 39 683 in 2022. This downward trend was captured with high accuracy by several forecasting approaches, particularly the ensemble model.

### 3.7 Model Validation Performance for Incidence

Validation performance metrics for incidence forecasting models are summarized in Table 3 and illustrated in Figure 3. The ensemble model demonstrated the highest predictive accuracy, achieving a Root Mean Square Error (RMSE) of 248, Mean Absolute Error (MAE) of 201, and Mean Absolute Percentage Error (MAPE) of 0.48% during the 2018–2022 validation period. Among individual models, ARIMAX performed best (MAPE 1.38%), followed by polynomial regression (MAPE 2.86%) and XGBoost (MAPE 3.20%). Traditional univariate ARIMA and Random Forest models showed comparatively poorer performance, with MAPE values exceeding 7%. *R* ^2^ values for incidence models demonstrated that only the ensemble approach achieved meaningful predictive accuracy during the validation period (*R* ^2^ = 0.51), while individual models produced negative *R* ^2^ values ranging from −1.13 (ARIMAX) to −68.51 (ARIMA).

**TABLE 3:** Incidence model validation performance (2018–2022).

Model	RMSE	MAE	MAPE (%)	*R* ^2^
Ensemble	247.55	201.32	0.48	0.51
ARIMAX	607.92	554.42	1.38	−1.13
Polynomial	1546.30	1147.99	2.86	−12.78
XGBoost	1575.13	1286.28	3.20	−15.45
ETS	1878.84	1619.00	4.03	−19.34
DLM	2382.45	2239.05	5.55	−31.71
Random Forest	3090.91	3026.77	7.49	−54.05
ARIMA	3473.01	3113.54	7.73	−68.51

*Note:* Negative *R* ^2^ values are expected in short validation windows containing structural turning points. In this setting, *R* ^2^ compares model predictions against the validation‐period mean, which is an unstable benchmark when the outcome reverses direction. MAPE provides a more informative measure of predictive accuracy and was used as the primary model selection criterion.

Abbreviations: MAE, mean absolute error; MAPE, mean absolute percentage error; *R* ^2^, coefficient of determination; RMSE, root mean square error.

**FIGURE 3:**
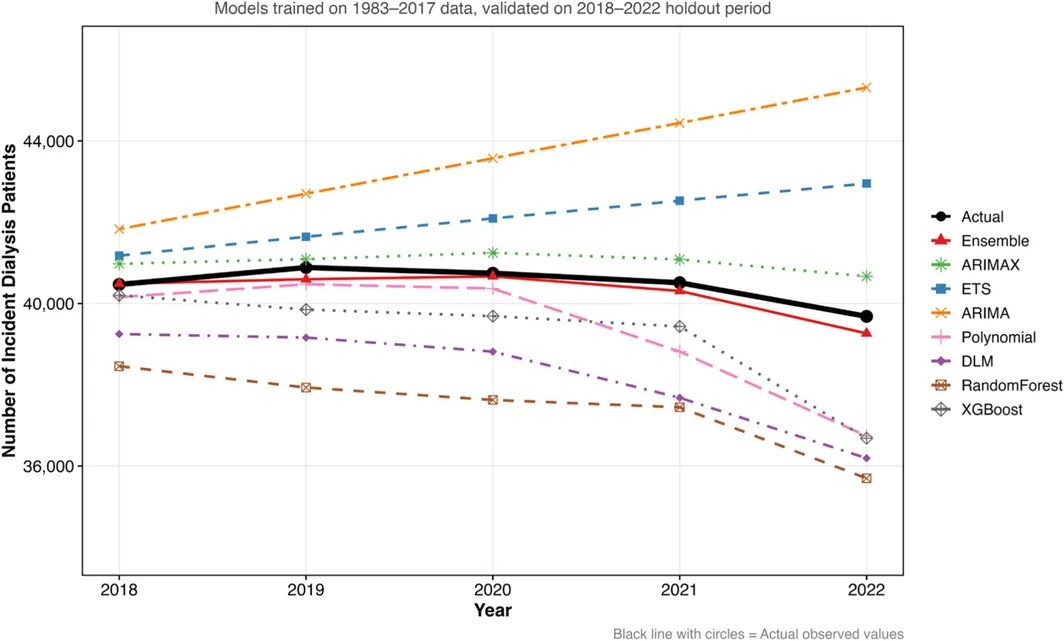
Incidence model validation performance (2018–2022).

Inverse‐MAPE weighting assigned the largest contribution to the ARIMAX model (34.9%), followed by polynomial regression (16.8%), XGBoost (15.0%), and ETS (11.9%), while ARIMA and Random Forest contributed minimally to the ensemble forecast.

### 3.8 Incidence Projections to 2050

Projected dialysis incidence trajectories diverged substantially across models over the long‐term forecast horizons, as seen in Figure 4 (2023–2050). The ensemble model projected a continued gradual decline in annual dialysis incidence, from 38 731 new patients in 2023 to 29 983 in 2050, corresponding to a 24.4% reduction relative to 2022 levels.

**FIGURE 4:**
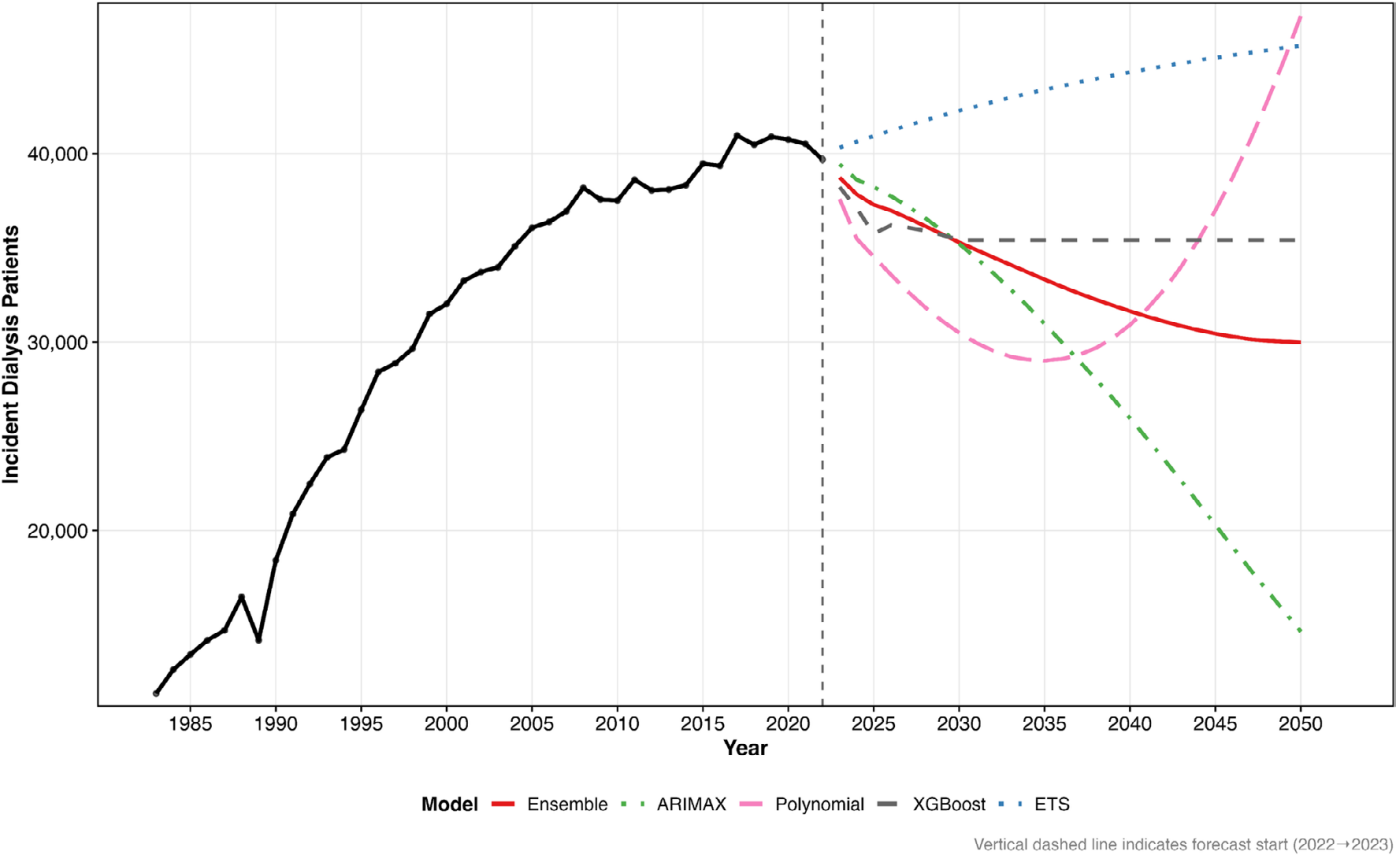
Projected incidence to 2050.

Among individual models, ARIMAX projected the most pronounced decline, with incident cases decreasing to 14 632 by 2050. In contrast, the ETS model projected a steady increase in incidence, reaching 45 729 by 2050, while the polynomial model produced a trend reversal with increasing incidence after the mid‐2030s. Among the models shown, the XGBoost model generated a nearly flat long‐term projection, consistent with the general tendency of machine‐learning approaches in this dataset. Although not shown in Figure 4, Random Forest similarly generated a flat trajectory.

Key projected incidence estimates from the ensemble model are presented in Table 4.

**TABLE 4:** Projected dialysis incidence (2025–2050).

Year	ARIMAX	POLYNOMIAL	XGBOOST	ETS	Ensemble
2025	38 224	34 523	35 747	40 928	37 284
2030	35 207	30 490	35 420	42 274	35 286
2035	30 964	29 005	35 420	43 391	33 316
2040	25 955	30 912	35 420	44 319	31 638
2045	20 287	36 992	35 420	45 089	30 443
2050	14 632	47 316	35 420	45 729	29 983

## 4 Discussion

### 4.1 Principal Findings

In 40 years of nationwide registry data, it was observed that dialysis prevalence in Japan reached an apparent peak in 2021 and declined modestly in 2022, although the contribution of pandemic‐related excess mortality to this inflection cannot be fully separated from underlying demographic trends at this stage. When extrapolated to 2050, forecasts diverged substantially across models. Approaches incorporating demographic covariates generally projected stabilization or decline in prevalence, whereas purely extrapolative or trend‐driven models suggested continued growth. A performance‐weighted ensemble model, which demonstrated the best out‐of‐sample accuracy, projected a gradual decline in dialysis prevalence to approximately 322 000 patients by 2050, alongside a sustained reduction in annual dialysis incidence.

These findings highlight that future dialysis burden in Japan is increasingly shaped by demographic forces rather than growth in incident disease alone. Declining incidence, combined with high competing mortality in an aging population, appears sufficient to offset gains in dialysis survival over the coming decades.

### 4.2 Comparison With Previous Projections

Our results strongly align with the seminal projection by Nakai and Wakasugi et al. [6, 12], who respectively projected that the prevalence would peak in 2021 and anticipated total incidence rates with less than 5% errors. Importantly, our analysis extends those projections by incorporating an additional decade of observed data and by formally comparing multiple forecasting strategies.

Earlier projection studies largely relied on regression‐based or demographic models [6, 9, 10], whereas our results demonstrate that ensemble approaches, when anchored to recent validation performance, can offer more balanced long‐term projections. Models such as ETS and univariate ARIMA, while performing reasonably in short‐term validation, tended to extrapolate historical growth patterns without adequately accounting for population contraction. In contrast, demographic models (ARIMAX and DLM) captured incidence decline more accurately but produced widely divergent long‐term prevalence trajectories depending on assumptions about survival and age effects.

### 4.3 International Context

However, the projected decline in dialysis incidence observed in Japan may not mirror trends in other high‐income countries. In the United States, end‐stage renal disease incidence prevalence relatively stabilized between 2019 and 2021; however, despite that an all‐time high of 808 536 prevalent ESRD patients were reported [7]. Similarly United Kingdom has reported their highest prevalence to date in 2023 [8]. However, a similar fall in incidence has been seen in Malaysia between 2022 to 2024, another Asian country that has historically had a high dialysis population [13]. Taiwan, the country with the highest per million dialysis prevalence globally, has also reported a slight decrease in prevalence between 2021 and 2022 [14]. However, the changes between these years must be interpreted cautiously as there might have been unexpected amounts of mortality in prevalent dialysis populations during the COVID‐19 pandemic [15]. Furthermore, our findings may not be generalizable to countries at different stages of demographic transition. For example, projections for Türkiye indicate that both RRT prevalence and incidence are expected to increase through 2040 [16].

Japan appears to be at the forefront of a transition in dialysis, possibly owing to its rapidly shrinking population and exceptionally high median age among dialysis patients; however, it remains unknown whether this trend will be followed by other countries or if it will remain specific to Japan, whereas most nations' dialysis populations continue to rise.

### 4.4 Evolving Etiology and Conservative Kidney Management

An important clinical factor not captured by our aggregate models is the changing composition of primary kidney diseases leading to dialysis initiation. In Japan, the proportion of incident dialysis patients with nephrosclerosis has been steadily increasing, reflecting both population aging and improved detection of hypertensive kidney disease [4]. Nephrosclerosis predominantly affects elderly patients, who tend to have shorter dialysis survival and higher competing mortality. This aetiological shift may partly explain the observed deceleration in net prevalence growth, as new patients initiated for nephrosclerosis contribute to incidence but exit the prevalent pool relatively quickly through death. Future disaggregated analyses by primary renal diagnosis would help quantify this effect.

Equally important is the growing adoption of conservative kidney management (CKM), also termed non‐dialytic supportive care, particularly among elderly Japanese patients with multiple comorbidities and limited life expectancy. Although systematic national data on CKM uptake in Japan remain sparse, clinical practice has increasingly favored shared decision‐making around dialysis initiation, and part of the observed decline in dialysis incidence may reflect deliberate decisions to forgo or defer RRT rather than a reduction in the underlying burden of advanced CKD [17]. This factor is not explicitly modeled in our projections and represents a key source of uncertainty: if CKM adoption accelerates, actual future incidence could decline faster than projected; conversely, if societal or clinical norms shift back toward universal dialysis initiation, incidence may remain higher than the ensemble forecast suggests.

### 4.5 Methodological Implications

Our findings also carry important methodological implications for long‐term health‐care forecasting. Machine‐learning models, including Random Forest and XGBoost, performed poorly in both prevalence and incidence forecasting, despite their flexibility. This likely reflects the limited sample size inherent to annual national time series and the dominance of slow‐moving demographic drivers that are better captured by structured statistical models [18]. Similar limitations of machine‐learning approaches have been noted in other chronic disease studies and end‐point predictions; revealing that the success of machine learning algorithms are highly dependent on the type and amount of data as well as modeling approach [19, 20]. More broadly, our results illustrate the limited utility of complex machine‐learning methods in small annual epidemiological datasets. With only 35 training observations, Random Forest and XGBoost lack the sample size needed to learn generalisable non‐linear patterns and instead risk fitting noise. Although regularization and tree‐depth constraints were applied (see Methods), these safeguards cannot fully compensate for the fundamental data scarcity. These models may be more appropriate in settings where higher‐resolution data (e.g., monthly or patient‐level records) are available.

The ensemble approach mitigated these weaknesses by weighting models according to empirical validation performance during a structurally informative period. Notably, while MAPE values for individual models remained within acceptable ranges (1%–10%), *R* ^2^ values were uniformly negative, indicating that no single model could adequately capture the structural break in dialysis trends during 2018–2022. This finding underscores a critical limitation of traditional time‐series approaches when applied to epidemiological transitions: models trained on decades of unidirectional growth struggle to identify inflection points in real time. The positive *R* ^2^ achieved by the ensemble (0.58 for prevalence, 0.51 for incidence) demonstrates that combining diverse modeling paradigms, each capturing different aspects of the underlying dynamics, can yield predictions that outperform any individual approach, particularly during periods of structural change. This strategy yielded projections that avoided both implausible exponential growth and extreme demographic pessimism, positioning the ensemble as a pragmatic tool for policy planning under uncertainty.

However, the substantial divergence among individual models visible in Figures 2 and 4 warrants further comment. Models such as ETS and ARIMA, which extrapolate historical trends without demographic input, project continued growth because they are unable to incorporate the structural effect of Japan's shrinking population. At the opposite extreme, the DLM amplifies age‐related effects over longer horizons, producing implausibly high estimates. The ensemble tempers these extremes by assigning higher weight to models that performed well during the validation period, which included the critical turning point. In effect, the ensemble functions as an empirical hedge against structural model mis‐specification: it does not resolve the underlying uncertainty about which model assumptions are correct, but it produces a forecast that is more robust to any single model being wrong. Users of these projections should therefore interpret the spread across individual models as a measure of structural uncertainty and the ensemble as a probability‐weighted central estimate.

### 4.6 Policy and Planning Implications

From a health‐system perspective, the projected stabilization or decline in dialysis prevalence does not imply reduced burden. The dialysis population in Japan will likely continue to age, with important implications for comorbidity burden, health‐care utilization, staffing needs, and costs [21, 22]. Furthermore, declining incidence may coexist with longer treatment durations per patient, reinforcing the need for sustained capacity planning.

These projections underscore the importance of continued investment in CKD prevention, conservative kidney care, and transplantation, alongside adaptation of dialysis services to an older, more complex patient population. In particular, the widespread adoption of kidney‐protective therapies, including SGLT2 inhibitors and GLP‐1 receptor agonists, may further reduce future dialysis incidence beyond what historical trends capture. Because these agents were introduced or expanded during or after our training period, their full effect is not reflected in the models, and actual incidence could decline more rapidly than projected if uptake continues to increase [23, 24]. Japan's experience may also provide valuable lessons for other aging societies approaching similar demographic thresholds.

### 4.7 Strengths and Limitations

The principal strength of this study lies in the use of four decades of high‐quality nationwide registry data and the explicit validation of competing models against a recent structural turning point.

The present study has several limitations that should be considered when interpreting these projections.

First, our analysis relies on aggregate annual data from a national registry. While the JRDR is exceptionally comprehensive, the use of macro‐level data precluded stratified projections by sex, geographical region, or specific primary causes of kidney failure (such as diabetic nephropathy versus glomerulonephritis), which may follow distinct trajectories.

Second, the “peak” in dialysis prevalence observed in 2021 and the subsequent decline in 2022 must be interpreted with caution. These years coincided with the COVID‐19 pandemic, which caused significant excess mortality among maintenance dialysis patients globally [15]. Consequently, it remains unclear to what extent the observed 2021 peak reflects a genuine structural shift driven by demographic forces, a transient mortality shock attributable to the pandemic, or a combination of both. At this stage, it is more appropriate to describe the data as suggesting a potential shift toward a downward trend rather than to conclude definitively that a post‐peak phase has been entered. To address this limitation, we performed sensitivity analyses comparing model performance during the pre‐pandemic period (2018–2019) with the full validation window (2018–2022). The relative ranking of models remained stable across both periods, with the Ensemble approach showing remarkable robustness—achieving a MAPE of 0.71% pre‐COVID and 0.62% during the pandemic period. However, several individual models, particularly ARIMA and other approaches without demographic covariates, showed substantial performance degradation during 2020–2022, suggesting that the pandemic introduced structural discontinuities that pure time‐series approaches struggled to capture. These findings underscore that our projections beyond 2022 should be interpreted as conditional forecasts assuming post‐pandemic dynamics return to pre‐COVID trajectories, rather than definitive predictions. Future studies incorporating additional years of post‐pandemic data will be essential to validate whether the 2021 peak represents a true inflection point or a pandemic‐related artifact.

Third, our models do not explicitly account for future shifts in clinical practice, healthcare policy, or the impact of emerging therapies. For instance, the widespread adoption of SGLT2 inhibitors and GLP‐1 receptor agonists, whose renal protective effects have been established in large‐scale trials, may further slow CKD progression and reduce future dialysis initiation rates beyond what is captured by historical trends. Similarly, the growing practice of conservative kidney management, particularly among elderly patients with a significant comorbidity burden, may explain part of the observed decline in incidence, rather than a true reduction in advanced CKD burden. Neither of these factors is explicitly modeled, and both represent important sources of directional uncertainty in our projections.

Finally, the poor performance of machine‐learning models (Random Forest and XGBoost) in our validation phase highlights the challenges of applying complex, non‐linear algorithms to relatively small annual datasets, whereas the negative *R* ^2^ values observed for individual models during the validation period highlight the inherent difficulty of forecasting epidemiological turning points and suggest that model performance metrics derived from periods of stable trends may not generalize to periods of structural change. While the ensemble approach mitigated this by weighting models based on empirical performance, all long‐term forecasts are inherently uncertain and should be viewed as scenario‐based estimates rather than definitive predictions.

## 5 Conclusions

In conclusion, dialysis prevalence in Japan shows signs of a potential shift toward a sustained downward trend, with future trajectories likely to be increasingly governed by demographic contraction rather than rising incidence. Ensemble forecasting grounded in empirical validation suggests a conditional projection of gradual decline in dialysis burden through 2050, assuming post‐pandemic dynamics return to pre‐COVID trajectories, although substantial uncertainty remains across modeling approaches. Japan's experience offers a critical reference point for other aging societies and highlights the need for adaptive, demographically informed planning of kidney care services.

## Funding

The authors have nothing to report.

## Ethics Statement

This study used publicly available data and did not involve human participants, patient data, or clinical intervention. Accordingly, ethical approval from an Institutional Review Board and informed consent were not required.

## Conflicts of Interest

The authors declare no conflicts of interest.

## Supporting information


**Table S1:** Ensemble model component weights.
**Table S2:** COVID‐19 sensitivity analysis: Prevalence model performance by validation period.
**Table S3:** COVID‐19 sensitivity analysis: Incidence model performance by validation period.
**Figure S1:** COVID‐19 sensitivity analysis: Model performance by validation period.
**Figure S2:** Change in model performance during COVID‐19 period.

## Data Availability

The data reported here have been provided by the Japanese Society for Dialysis Therapy (JSDT). The interpretation and reporting of these data are the responsibility of the authors and in no way should be seen as an official policy or interpretation of the JSDT.
