# Supplementary material for: Forecasting the Dialysis Burden in Japan: Validation‐Based Projections of Prevalence and Incidence Through 2050
Source: Ther Apher Dial. 2026 Jul 6;30(6):925–34. doi: 10.1002/1744-9987.70183 (PMC13738047; doi:10.1002/1744-9987.70183)
Supplement: Supplementary file 1 [file TAP-30-925-s001.docx]

**SUPPLEMENTARY MATERIALS**

Forecasting the Dialysis Burden in Japan: Validation-Based Projections

Of Prevalence And Incidence Through 2050

**Supplementary Table S1.** Ensemble Model Component Weights

| **Model** | **Prevalence Weight (%)** | **Incidence Weight (%)** |
| --- | --- | --- |
| ETS | 41.80 | 10.18 |
| DLM | 17.43 | 7.39 |
| ARIMAX | 11.93 | 29.74 |
| Polynomial | 11.42 | 14.35 |
| ARIMA | 11.10 | 5.31 |
| XGBoost | 4.23 | 27.54 |
| RandomForest | 2.09 | 5.48 |

*Note: Weights were calculated as the inverse of each model's Mean Absolute Percentage Error (MAPE) during validation, normalized to sum to 100%. Higher weights indicate better validation performance. For prevalence, ETS dominated the ensemble (41.8%); for incidence, ARIMAX (29.7%) and XGBoost (27.5%) received the highest weights.*

**Supplementary Table S2.** COVID-19 Sensitivity Analysis: Prevalence Model Performance by Validation Period

| **Model** | **Pre-COVID MAPE (%)** | **COVID MAPE (%)** | **Full Period MAPE (%)** | **Δ MAPE (%)** | **Ranking Change** |
| --- | --- | --- | --- | --- | --- |
| Ensemble | 0.71 | 0.62 | 0.66 | -0.08 | Stable (1→1) |
| ETS | 0.09 | 1.67 | 1.03 | +1.58 | 1→2 |
| DLM | 0.57 | 3.75 | 2.47 | +3.18 | 2→4 |
| ARIMAX | 1.00 | 5.35 | 3.61 | +4.36 | 3→6 |
| Polynomial | 2.34 | 4.72 | 3.77 | +2.38 | 5→5 |
| ARIMA | 1.36 | 5.55 | 3.88 | +4.19 | 4→7 |
| XGBoost | 6.49 | 12.62 | 10.17 | +6.13 | 6→3 |
| RandomForest | 14.43 | 24.73 | 20.61 | +10.30 | 7→8 |

*Abbreviations: MAPE, Mean Absolute Percentage Error; Δ, change from pre-COVID to COVID period. Pre-COVID period: 2018-2019 (n=2 years); COVID period: 2020-2022 (n=3 years). Negative Δ indicates improved performance during COVID period. Ranking Change shows position in the pre-COVID period → COVID period rankings based on MAPE. The Ensemble model was the only approach showing improved or stable performance during the pandemic period.*

**Supplementary Table S3.** COVID-19 Sensitivity Analysis: Incidence Model Performance by Validation Period

| **Model** | **Pre-COVID MAPE (%)** | **COVID MAPE (%)** | **Full Period MAPE (%)** | **Δ MAPE (%)** | **Ranking Change** |
| --- | --- | --- | --- | --- | --- |
| Ensemble | 0.34 | 0.57 | 0.48 | +0.23 | Stable (1→1) |
| ARIMAX | 0.88 | 1.71 | 1.38 | +0.83 | 3→2 |
| XGBoost | 0.51 | 2.15 | 1.49 | +1.64 | 2→3 |
| Polynomial | 0.89 | 4.18 | 2.86 | +3.29 | 4→4 |
| ETS | 1.80 | 5.51 | 4.03 | +3.71 | 5→5 |
| DLM | 3.62 | 6.84 | 5.55 | +3.22 | 6→6 |
| RandomForest | 6.10 | 8.42 | 7.49 | +2.32 | 7→7 |
| ARIMA | 3.90 | 10.28 | 7.73 | +6.38 | 8→8 |

*Abbreviations: MAPE, Mean Absolute Percentage Error; Δ, change from pre-COVID to COVID period. Pre-COVID period: 2018-2019 (n=2 years); COVID period: 2020-2022 (n=3 years). The ARIMA model showed the largest performance degradation (+6.38 percentage points), reflecting its inability to capture pandemic-induced structural changes without external covariates.*

**Supplementary Figure S1.** COVID-19 Sensitivity Analysis: Model Performance by Validation Period

*Figure legend: Grouped bar chart comparing Mean Absolute Percentage Error (MAPE) across three validation periods: pre-COVID (2018-2019, green), COVID-affected (2020-2022, red), and full validation period (2018-2022, blue). Panel A shows prevalence models; Panel B shows incidence models. The shaded region highlights the Ensemble model, which maintained the most stable performance across periods. Horizontal dashed line indicates the 5% MAPE threshold for acceptable model performance.*

**Supplementary Figure S2.** Change in Model Performance During COVID-19 Period

*Figure legend: Lollipop chart showing the change in MAPE (Δ MAPE) from the pre-COVID period (2018-2019) to the COVID-affected period (2020-2022). Green indicates improved performance during COVID; red indicates degraded performance. Panel A shows prevalence models; Panel B shows incidence models. The Ensemble model for prevalence was the only approach showing improved performance during the pandemic period (Δ MAPE = -0.08%), while ARIMA showed the largest degradation for both outcomes.*
